# Chronic recurrent multifocal osteomyelitis manifested as painful clavicular swelling: a case report

**DOI:** 10.1186/1756-0500-7-786

**Published:** 2014-11-05

**Authors:** Markus Bleckwenn, Bernd Sommer, Klaus Weckbecker

**Affiliations:** Department of General Practice and Family Medicine, University Medical Center Friedrich-Wilhelm University Bonn, Sigmund-Freud-Str. 25, 53127 Bonn, Germany; Practice for Radiology and Nuclear Medicine, Am Cura-Krankenhaus, Schülgenstr. 15a, 53604 Bad Honnef, Germany

**Keywords:** Osteomyelitis, CRMO, Clavicular swelling, Hyperostosis, Diagnostic imaging, Nonbacterial inflammation

## Abstract

**Background:**

Chronic recurrent multifocal osteomyelitis is a form of non-bacterial osteomyelitis which occurs primarily in childhood. In some cases painful bone swelling occurs. After a malignancy has been ruled out, antibiotic therapy is often started to treat the osteomyelitis. The course of this benign disease is self-limiting and is not positively affected by the antibiotic therapy.

**Case presentation:**

A 14-year-old German girl from South Africa came to the surgery with painful swelling in the right clavicle. The condition had first appeared two months earlier. The patient was unable to identify a trigger. In addition, the patient exhibited painless swelling in the area of the 5^th^ metatarsal of the left foot. Chronic recurrent multifocal osteomyelitis was diagnosed based on characteristic clinical symptoms and imaging. Treatment with ibuprofen had caused the symptoms to regress rapidly.

**Conclusion:**

The case demonstrates to general practitioners and other clinicians that a prolonged administration of antibiotics can be prevented by means of a comprehensive diagnostic procedure for possible bacterial osteomyelitis.

## Background

Chronic recurrent multifocal osteomyelitis (CRMO) is a rare form of endogenic aseptic inflammation of the bone marrow of unknown aetiology, which takes a sub-acute or primary chronic course. Since the condition is often accompanied by other autoimmune disorders, it is commonly considered that CRMO is in its origin caused by autoimmune processes. CRMO was first described in 1972 by Giedeon *et al*. and affects girls more often than boys with a peak age of 10 years [1]. All bones can be affected. The clavicle is a localisation in approximately 9% of all osteomyelitis cases among paediatric patients [2].

Patients complain of local pain which increases under stress, but are always in a good general state of health. About one-tenth of the patients develop a pronounced hyperostosis in the seat of infection [2]. The illness normally takes a benign and self-limiting course, whereby the average duration is approximately two years. However, around 20% of the patients suffer a longer course of illness with frequent relapses [3]. Treatment of CRMO is primarily symptomatic. In addition non-steroidal anti-inflammatory drugs are employed. Even in the absence of pathogen detection, antibiotic treatment is initiated almost on a regular basis and often continued for weeks and months. However, this changes nothing in the natural course of the disease [2].

The diagnosis of CRMO is based on characteristic clinical symptoms, imaging and possibly histological findings. This case is presented to show how it is possible to diagnose a CRMO in a short time with targeted diagnostics, thus preventing extensive and invasive diagnosis and therapy.

## Case presentation

A German girl visited the surgery during consultation hours with swelling in the right clavicle. The condition had first appeared two months earlier. The girl was not aware of any specific trigger, although at that time she had gone hiking for two weeks with a backpack and had originally attributed the pain to excessive exertion. The pain disappeared after one treatment with ibuprofen. However, the swelling remained unchanged. On questioning, her mother admitted that the girl had swelling in the region of the 5th metatarsal of the left foot, which however caused her no pain. The patient said she had suffered neither weight loss nor B symptoms. She played sports regularly and enjoyed pain-free mobility. Long-standing asthma was treated twice daily with budesonide and formoterol.

**Figure 1:**
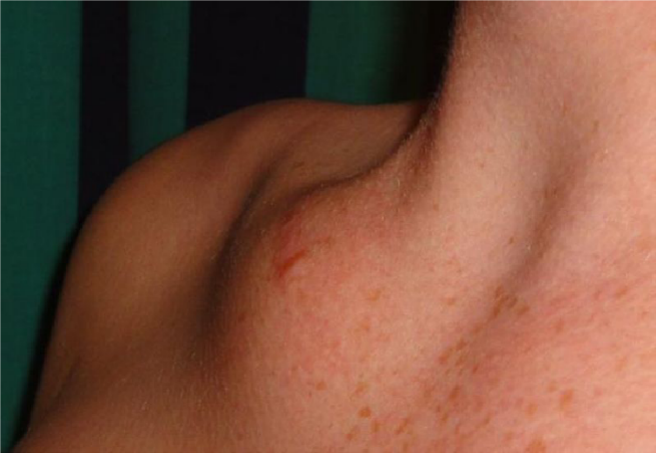
**Swelling of the right clavicle – the abnormality has not changed in appearance since the first contact with the patient.**

The examination revealed a distinct swelling of the clavicle (Figure 1). On palpation it proved to be a hard, non-displaceable lesion measuring 8 × 5 cm. In comparison with the left clavicle a notably higher temperature could be detected in the swollen region. A systematic examination yielded no other visible findings. In the presence of clavicular swelling, it is especially important to rule out a malignancy (Table 1).

**Table 1:** **Differential diagnosis of clavicular swelling**

•	Bacterial osteomyelitis
•	Langerhans cell histiocytosis
•	Ewing’s sarcoma
•	Osteosarcoma
•	Leukemic bone marrow infiltration
•	Bone metastases (also from an osteosarcoma)
•	Chronic recurrent multifocal osteomyelitis (CRMO)

**Figure 2:**
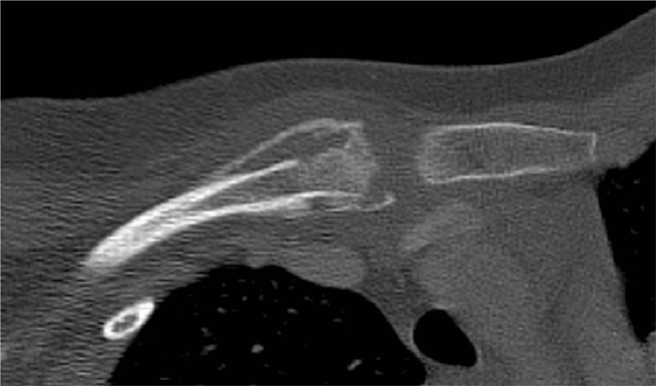
**Computed tomography scan of the clavicle with cortical osteolysis and bowl-shaped periosteal hyperostosis.**

Because of the typical symptoms and the fact that time for examination was limited to one day before the patient was due to depart for South Africa, it was decided in the assessment of the case to forego a conventional x-ray photograph of the clavicle.To reduce the potential exposure to radiation, we first performed magnetic resonance imaging (MRI) of the clavicle and the upper superior thoracic aperture. This showed evidence of a pathological bone marrow signal of the medial clavicle 5 cm in length. Involvement of the articular capsule was found, albeit without any evidence of an effusion. The conducted MRI could not rule out the presence of a malignant tumour or bacterial osteomyelitis. Thus, despite the moderate exposure to radiation, it was subsequently deemed necessary to perform a computed tomography (CT) scan of the thorax using an acceptably adapted dose for paediatric purposes (Figure 2).

The CT scan detected osteolysis in the left and right dorsal to the tenth thoracic vertebra. From the perspective of the differential diagnosis, the presence of several infectious foci made bacterial osteomyelitis or primary malignant bone marrow tumours highly unlikely.

In the laboratory borderline monocyte levels of 6.1% were observed, as well as borderline serum sodium levels of 144 mmol/litre and an erythrocyte sedimentation rate of 22 mm in the first hour. All other parameters were normal and age-appropriate for a paediatric patient. After the examinations, the patient had to return to South Africa where a bone marrow biopsy was taken and the adjacent swollen lymph node (benign, reactive) was simultaneously removed. Bone scintigraphy was also performed.

The bone marrow biopsy showed active, sub-acute osteomyelitis. Additionally, increased bone density and marrow fibrosis were observed, consistent with the pattern of an earlier bone fracture.

In the literature, ossifying elements are documented in histopathological examinations of CRMO [4]. In this case there was no anamnestic evidence that the findings had a traumatic aetiology.

The skeletal scintigraphy was especially intended to detect any asymptomatic foci of infection. Alternatively, a full-body MRI could have been performed. There was evidence of osteomyelitis in the right proximal collarbone and at the base of the 5th metatarsal of the left foot. In addition increased activity was observed in the left hip and the right ankle.

In South Africa the patient has so far received no specific treatment. The clavicular swelling has remained unchanged to this day. It now only sporadically causes pain. The patient has not needed to take any analgesics since her visit to Germany.

One possible complication could be the destruction or a pathological fracture of the thoracic vertebrae. Stagnation of the cosmetically bothersome hyperostosis of the clavicle is also possible. Schulz *et al*. reported their own experiences in treating a total of 5 patients between 7–17 years old [5]. These patients had initial complaints involving the ankles, the thoracic vertebrae, a rib and the clavicle. In all patients, for example, the diagnosis consisted of more osseous foci in the humerus or the femur. Therefore, it is recommended that the girl in question undergo further clinical examination, particularly if new symptoms arise. Four weeks after the start of a symptom plain radiography should be performed. Back pain or pelvic pain could be better clarified by MRI [2].

## Conclusions

After having ruled out a malignancy (laboratory, x-ray and scintigraphy), the tentative diagnosis of CRMO should have made it possible to forego additional diagnostic procedures. Although more extensive diagnostics were performed in the case under discussion, it was nevertheless possible to avoid lengthy antibiotic treatment.

## Consent

Written informed consent was obtained from the patient’s parents for publication of this Case Report and any accompanying images, as the patient is a minor. A copy of the written consent is available for review by the Editor-in-Chief of this journal.

## Abbreviations

CRMO: Chronic recurrent multifocal osteomyelitis
MRI: Magnetic resonance imaging
CT: Computed tomography.
